# Effects of Waterpipe Smoke Exposure on Experimentally Induced Chronic Kidney Disease in Mice

**DOI:** 10.3390/ijms25010585

**Published:** 2024-01-02

**Authors:** Sumaya Beegam, Suhail Al-Salam, Nur Elena Zaaba, Ozaz Elzaki, Badreldin H. Ali, Abderrahim Nemmar

**Affiliations:** 1 Department of Physiology, College of Medicine and Health Sciences, United Arab Emirates University, Al Ain P.O. Box 15551, United Arab Emirates; sumayab@uaeu.ac.ae (S.B.); elenazaaba@uaeu.ac.ae (N.E.Z.); ozazelzaki@uaeu.ac.ae (O.E.); 2 Department of Pathology, College of Medicine and Health Sciences, United Arab Emirates University, Al Ain P.O. Box 15551, United Arab Emirates; suhaila@uaeu.ac.ae; 3 Zayed Center for Health Sciences, United Arab Emirates University, Al Ain P.O. Box 15551, United Arab Emirates; 4 Independent Researcher, Lincoln, NE 68507, USA; alibadreldin@hotmail.com

**Keywords:** waterpipe smoke, chronic kidney disease, oxidative stress, DNA damage, inflammation

## Abstract

Tobacco smoking is an independent risk factor in the onset of kidney disease. To date, there have been no reports on the influence of waterpipe smoke (WPS) in experimentally induced chronic kidney disease (CKD) models. We studied the effects and mechanisms of actions of WPS on a mouse model of adenine-induced CKD. Mice fed either a normal diet, or an adenine-added diet and were exposed to either air or WPS (30 min/day and 5 days/week) for four consecutive weeks. Plasma creatinine, urea and indoxyl sulfate increased and creatinine clearance decreased in adenine + WPS versus either WPS or adenine + saline groups. The urinary concentrations of kidney injury molecule-1 and adiponectin and the activities of neutrophil gelatinase-associated lipocalin and N-acetyl-β-D-glucosaminidase were augmented in adenine + WPS compared with either adenine + air or WPS groups. In the kidney tissue, several markers of oxidative stress and inflammation were higher in adenine + WPS than in either adenine + air or WPS groups. Compared with the controls, WPS inhalation in mice with CKD increased DNA damage, and urinary concentration of 8-hydroxy-2-deoxyguanosine. Furthermore, the expressions of nuclear factor κB (NF-κB) and mitogen-activated protein kinases (MAPKs) (ERK and p38) were elevated in the kidneys of adenine + WPS group, compared with the controls. Likewise, the kidneys of adenine + WPS group revealed more marked histological tubular injury, chronic inflammation and interstitial fibrosis. In conclusion, WPS inhalation aggravates kidney injury, oxidative stress, inflammation, DNA damage and fibrosis in mice with adenine-induced CKD, indicating that WPS exposure intensifies CKD. These effects were associated with a mechanism involving NF-κB, ERK and p38 activations.

## 1. Introduction

Waterpipe smoke (WPS) consumption has swiftly become a worldwide tobacco epidemic which has received wide acceptance, mostly among the youth. The latter has been associated with the use of flavored and sweetened waterpipe tobacco and the misconceptions that it is safer and less addictive than cigarette smoke (CS) [1,2,3]. WPS comprises various carcinogens that include polycyclic aromatic hydrocarbons, naphthylamines, tobacco-specific nitrosamines, and primary aromatic amines, together with CO carbonyls (for example acetaldehyde, formaldehyde or acrolein) [4,5]. A session of WPS may last between 30 and 90 min. It has been reported that compared with CS, WPS is linked with greater CO and tar, comparable nicotine, and substantially more smoke exposure [4,5].

Tobacco smoking is considered an independent risk factor for the development of chronic kidney disease (CKD) in both patients with type 2 diabetes, and in the general population [6,7,8]. It is well known that inhaled pollutants including smoking can cause lung inflammation and spill-over of inflammatory cytokines into the systemic circulation which can affect distant organs such the kidney, and several toxicants can pass through the respiratory membrane and reach the systemic circulation [7,9]. Moreover, since the kidneys receive 20% of the cardiac output and a significant amount of blood is filtered, this makes the kidney exposed to concentrated toxic substances from smoking and air pollution [7]. Diseases of the kidneys are being recognized as a major public health problem, and cases of CKD have reached an epidemic level globally with an estimated prevalence of 13.4% [10].

The establishment of a causal relationship between WPS and CKD poses numerous difficulties, encompassing the failure to distinguish between the effects induced by WPS inhalation and the impact of other factors, e.g., exposure to CS or environmental toxicants [11]. Consequently, experimental work on the impact of WPS on CKD is pertinent and critically needed.

The data on the impact of WPS on the kidneys is still very scant. Recent works including ours, have reported that exposure to WPS induces the impairment of kidney function, inflammation and oxidative stress [12,13]. Epidemiological and clinical investigations indicate that the effects of air pollution in general (including tobacco smoking) are more pronounced in vulnerable subjects, such as those with CKD [7,8]. While some clinical and epidemiological studies have reported associations between WPS and CKD [14], to the best of our knowledge, no study has evaluated the impact of WPS in patients with established CKD and compared to that in “healthy” subjects who smoke waterpipe. Likewise, no experimental study has assessed the effects of WPS in an established model of CKD. The latter approach will provide biological plausibility for the effects of WPS on established CKD, and would mimic a situation of healthy subjects and patients with CKD smoking waterpipe. This is clinically relevant because it is well known that many patients live with nonchronic conditions for many years before they are diagnosed with chronic conditions [15]. This is especially true in the case of patients with CKD [15]. Globally, CKD affects >10% of the general population, amounting to >800 million persons [16]. More than half (51.8%) of adults in the USA had at least one out of ten of some diagnosed chronic conditions including weak or failing kidneys, and 27.2% of US adults had multiple chronic conditions [17]. CKD involves gradual loss of kidney function, and in its early stages, the patients might have no or few signs or symptoms for years until they begin displaying symptoms of kidney failure [15,17]. During that period the patients continue with their regular life style that may include using tobacco products (such as WPS). Since it is well known that smoking tobacco increases the risk factor for CKD [7,8], an investigation into the condition of CKD, or its progression, in experimental animals concomitantly exposed to WPS is considered important.

Therefore, using a well-established mouse model of CKD induced by adenine [18,19], we aimed here to explore the extent of this experimental CKD in mice would be aggravated by WPS inhalation, and the possible mechanisms underlying this effect.

## 2. Results

### 2.1. Body Weight, Water Intake and Urine Volume Following WPS Exposure in Mice with CKD

**Figure 1:**
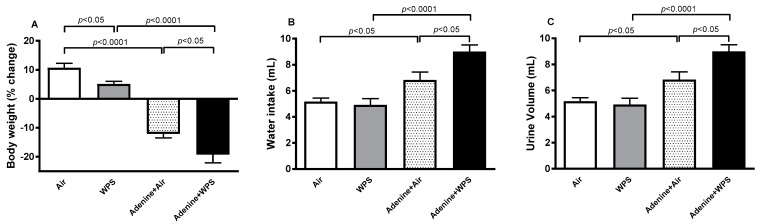
Body weight change (**A**), water intake (**B**) and urine volume (**C**) in mice exposed to either air or WPS for four weeks with or without adenine treatment to induce chronic kidney disease. Mean ± SEM (n = 6–8). The statistical analysis encompassed a one-way analysis of variance, with subsequent application of Holm–Sidak’s multiple comparisons test.

Compared with air group, the exposure to WPS induced a slight but a significant decrease in the % of body weight change (*p* < 0.05). Likewise, there was a substantial decrease in the % of body weight change in adenine + air group versus air group (*p* < 0.0001). Moreover, WPS exposure in the adenine-treated group induced a significant decrease in the % of body weight gain when compared with adenine + air (*p* < 0.05) and WPS (*p* < 0.0001) groups (Figure 1A). Both the water intake and urine volume were significantly increased in the adenine + WPS group when compared with adenine + air (*p* < 0.05) and WPS (*p* < 0.0001) groups (Figure 1B,C).

### 2.2. Urea, Creatine and Indoxyl Sulfate Concentrations in Plasma and Creatinine Clearance Following WPS Exposure in Mice with CKD

**Figure 2:**
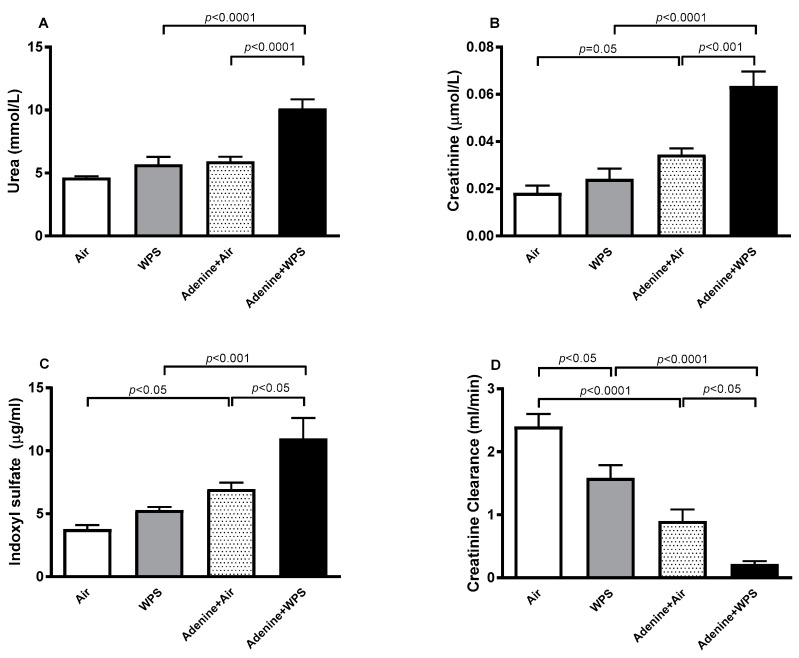
Plasma concentrations of urea (**A**), creatinine (**B**) and indoxyl sulfate (**C**), and creatinine clearance (**D**) in mice exposed to either air or WPS for four weeks with or without adenine treatment to induce chronic kidney disease. Mean ± SEM (n = 5–8). The statistical analysis encompassed a one-way analysis of variance, with subsequent application of Holm–Sidak’s multiple comparisons test.

As illustrated in Figure 2A–C, the plasma concentrations of urea, creatinine and the uraemic toxins indoxyl sulphate were significantly elevated adenine-treated mice exposed to WPS compared with adenine-treated mice exposed to air (*p* < 0.0001–*p* < 0.05) and those exposed to WPS (*p* < 0.0001–*p* < 0.001). On the other hand, the creatinine clearance was significantly decreased in adenine + WPS group compared with adenine + air (*p* < 0.05) and WPS (*p* < 0.0001) groups (Figure 2D).

### 2.3. Neutrophil Gelatinase- Associated Lipocalin (NGAL), Kidney Injury Molecule-1 (KIM-1), N-acetyl-β-D-glucosaminidase (NAG) and Adiponectin in Urine Following WPS Exposure in Mice with CKD

**Figure 3:**
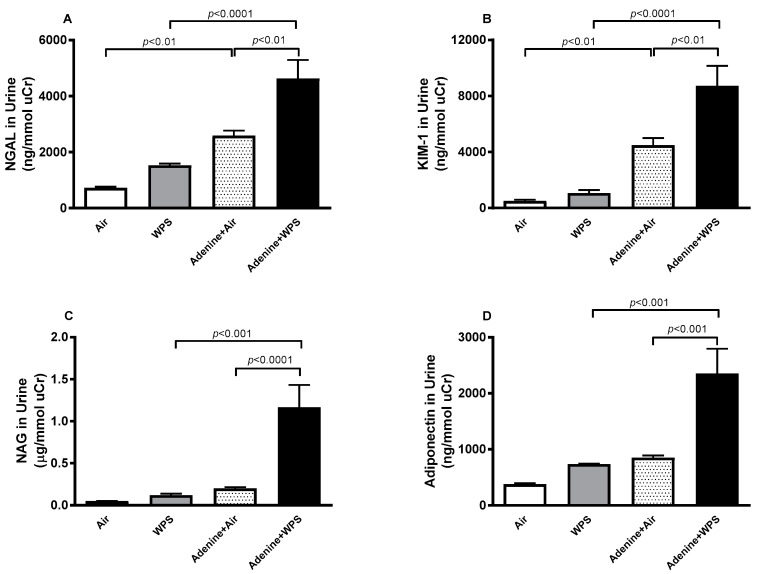
Neutrophil gelatinase-associated lipocalin (NGAL; (**A**)), kidney injury molecule-1 (KIM-1; (**B**)), N-acetyl-β-glucosaminidase (NAG; (**C**)) and adiponectin (**D**) levels in the urine of mice exposed to either air or WPS for 4 weeks with or without treatment with adenine to trigger chronic kidney disease. Data are normalized to urinary creatinine concentration and shown as Mean ± SEM (n = 6). The statistical analysis encompassed a one-way analysis of variance, with subsequent application of Holm–Sidak’s multiple comparisons test.

The concentrations of kidney injury markers NGAL and KIM-1 and the biomarker of proximal tubular damage in urine, NAG, and the marker for the progression of CKD adiponectin were all significantly increased adenine + WPS group when compared with adenine + air (*p* < 0.0001–*p* < 0.01) and WPS (*p* < 0.0001–*p* < 0.001) groups (Figure 3).

### 2.4. Tumor Necrosis Factor-α (TNFα), Interleukin (IL)−6 and IL-1β Concentrations in Kidney Tissue Following WPS Exposure in Mice with CKD

**Figure 4:**
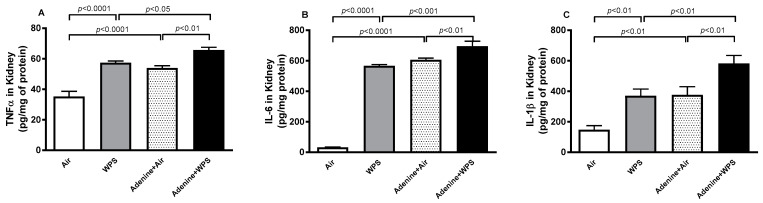
Tumor necrosis factor-α (TNFα; (**A**)), interleukin-6 (IL-6; (**B**)) and IL-1β (**C**) concentrations in kidney homogenates of mice exposed to either air or WPS for four weeks with or without adenine treatment to induce chronic kidney disease. Mean ± SEM (n = 6–8). The statistical analysis encompassed a one-way analysis of variance, with subsequent application of Holm–Sidak’s multiple comparisons test.

Figure 4 shows the concentrations of the proinflammatory cytokines TNFα, IL-6 and IL-1β in mice exposed to either air or WPS with or without adenine treatment. Compared with air-exposed group, WPS inhalation induced a significant increase in the concentrations of TNFα, IL-6 and IL-1β in the kidney tissue (*p* < 0.0001–*p* < 0.01). Likewise, the concentrations of these three cytokines in the kidney tissue were higher in the adenine + air group versus the air group (*p* < 0.01–*p* < 0.0001). Remarkably, the concentrations of TNFα, IL-6 and IL-1β in the kidney tissue of adenine + WPS group was significantly elevated when compared with adenine + air (*p* < 0.01) and WPS (*p* < 0.001–*p* < 0.05) groups.

### 2.5. TNFα, IL-6 and IL-1β Concentrations in Plasma Following WPS Exposure in Mice with CKD

**Figure 5:**
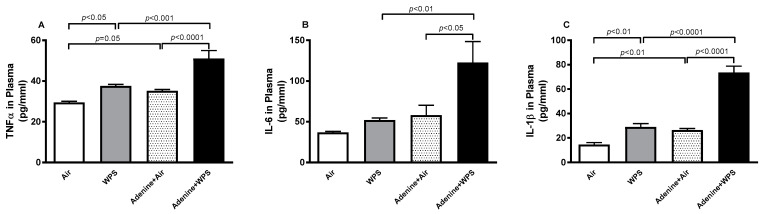
Tumor necrosis factor-α (TNFα; (**A**)), interleukin-6 (IL-6; (**B**)) and IL-1β (**C**) concentrations in plasma of mice exposed to either air or WPS for four weeks with or without adenine treatment to induce chronic kidney disease. Mean ± SEM (n = 8). The statistical analysis encompassed a one-way analysis of variance, with subsequent application of Holm–Sidak’s multiple comparisons test.

Figure 5 depicts the plasma concentrations of TNFα, IL-6, and IL-1β in mice exposed to either air or waterpipe smoke (WPS), with or without adenine treatment. Compared to the air-exposed group, WPS inhalation resulted in a significant rise in TNFα and IL-1β plasma concentrations (*p* < 0.01–*p* < 0.05). Similarly, the concentrations of these two cytokines in the adenine + air group were higher than in the air group (*p* < 0.01–*p* = 0.05). In contrast, the concentration of IL-6 in the WPS group compared to the air group, and adenine + air compared to air, showed insignificant increases. Notably, the concentrations of TNFα, IL-6, and IL-1β in the plasma of the adenine + WPS group were significantly elevated compared to adenine + air (*p* < 0.0001–*p* < 0.05) and WPS (*p* < 0.0001–*p* < 0.01) groups.

### 2.6. Lipid Peroxidation (LPO) and Reduced Glutathione (GSH) Concentrations and Superoxide Dismutase (SOD) Activity in Kidney Tissue Following WPS Exposure in Mice with CKD

**Figure 6:**
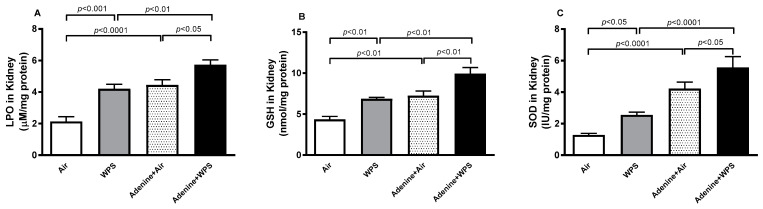
Concentrations of lipid peroxidation (LPO; (**A**)) and reduced glutathione (GSH; (**B**)) and activity of superoxide dismutase (SOD; (**C**)) in kidney homogenates of mice exposed to either air or WPS for four weeks with or without adenine treatment to induce chronic kidney disease. Mean ± SEM (n = 7–8). The statistical analysis encompassed a one-way analysis of variance, with subsequent application of Holm–Sidak’s multiple comparisons test.

Figure 6 shows that in comparison with air-exposed group, WPS inhalation induced a significant increase in the concentrations of LPO and GSH, and the activity of SOD (*p* < 0.001–*p* < 0.05). Similarly, the aforementioned markers of oxidative stress were all augmented in the adenine + air group compared with the air group (*p* < 0.0001–*p* < 0.01). In adenine + WPS, there was a significant elevation in the concentrations of LPO and GSH, and the activity of SOD in the kidney tissue compared with either adenine + air (*p* < 0.01–*p* < 0.05) or WPS (*p* < 0.0001–*p* < 0.01) group.

### 2.7. DNA Damage in Kidney Tissue and Urine Following WPS Exposure in Mice with CKD

**Figure 7:**
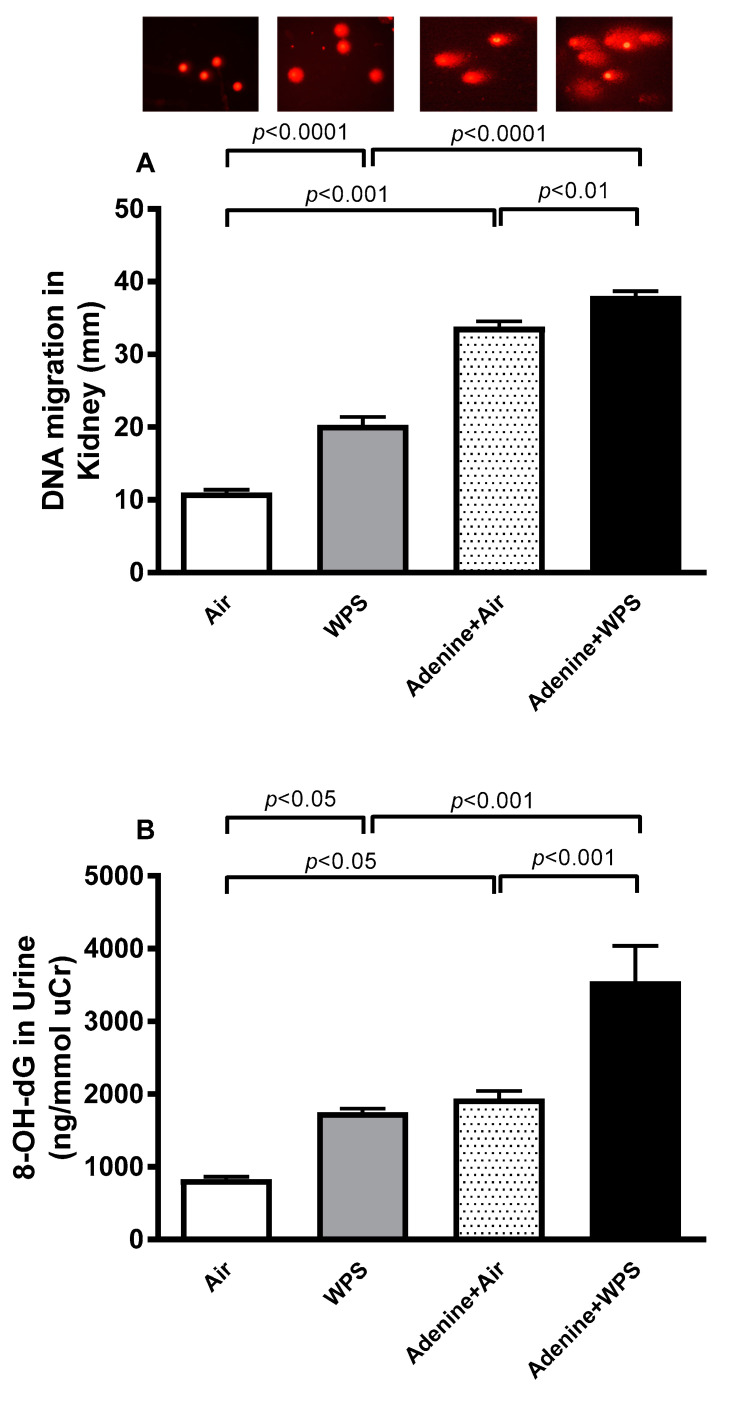
DNA damage (**A**) evaluated by COMET assay in kidney homogenate and representative images (magnification 40x), illustrating the quantification of the DNA migration, under alkaline conditions in mice exposed to either air or WPS for four weeks with or without adenine treatment to induce chronic kidney disease. Mean ± SEM (n = 5). Urinary concentration of 8-hydroxy-2′-deoxyguanosine (8-OHdG; (**B**)) in mice exposed to either air or WPS for four weeks with or without adenine treatment to induce chronic kidney disease. Data for 8-OH-dG are normalized to urinary creatinine concentration. Mean ± SEM (n = 6). The statistical analysis encompassed a one-way analysis of variance, with subsequent application of Holm–Sidak’s multiple comparisons test.

The DNA injury was assessed in the kidney tissue using a comet assay and in the urine by measuring 8-hydroxy-2′-deoxyguanosine (8-OH-dG), a marker of oxidative DNA damage (Figure 7). Both DNA migration in the kidney tissue, indicating DNA damage, and the concentration of 8-OH-dG in the urine were significantly increased in the WPS versus air (*p* < 0.0001–*p* < 0.05) and adenine + air versus air (*p* < 0.001–*p* < 0.05) groups. The latter effects were exacerbated in mice treated with adenine and exposed to WPS compared with either adenine + air (*p* < 0.001–*p* < 0.01) or WPS (*p* < 0.0001–*p* < 0.001) group.

### 2.8. Total and Phosphorylated (Phospho) Nuclear Factor κappa B (NF-κB) Expression in Kidney Tissue Following WPS Exposure in Mice with CKD

**Figure 8:**
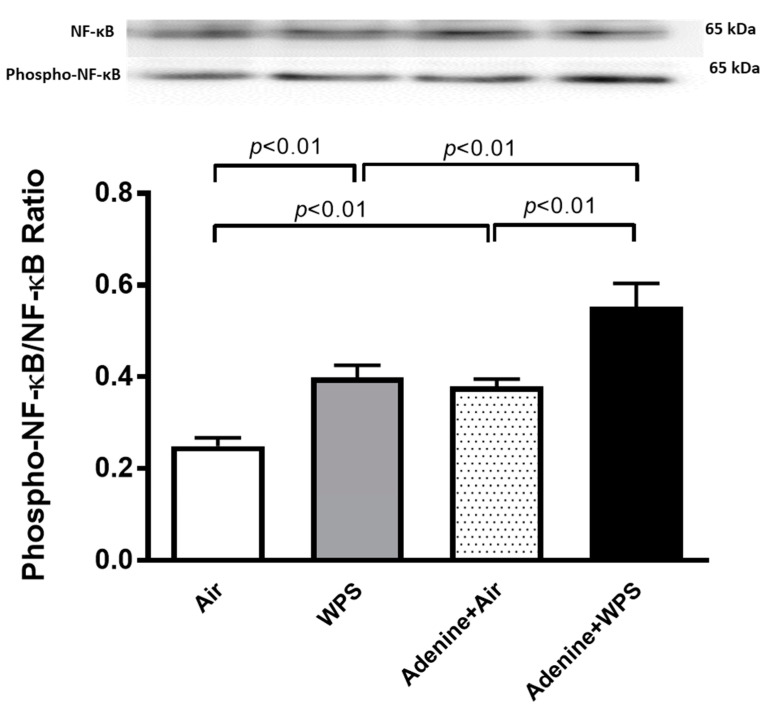
Western blot analysis of total and phosphorylated nuclear factor κappa B (phospho-NF-κB) expression in kidney homogenates of mice exposed to either air or WPS for four weeks with or without adenine treatment to induce chronic kidney disease. Mean ± SEM (n = 8). The statistical analysis encompassed a one-way analysis of variance, with subsequent application of Holm–Sidak’s multiple comparisons test.

The phospho-NF-κB/NF-κB in kidney tissue homogenates was increased in WPS group when compared with air-exposed group (*p* < 0.01). In the same way, the phospho-NF-κB/NF-κB was increased in adenine + air group when compared with air group (*p* < 0.01). Interestingly, the phospho-NF-κB/NF-κB was more increased in the adenine + WPS group when compared with the adenine + air (*p* < 0.01) and WPS (*p* < 0.01) groups (Figure 8).

### 2.9. Total and Phosphorylated (Phospho) Extracellular Signal-Regulated Kinase (ERK), c-Jun NH2-Terminal Kinase (JNK), and p-38 Expression in Kidney Tissue Following WPS Exposure in Mice with CKD

**Figure 9:**
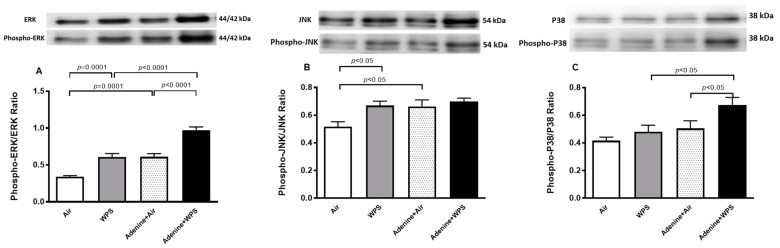
Western blot analysis of total and phosphorylated extracellular signal-regulated kinase (ERK, (**A**)), c-Jun NH2-terminal kinase (JNK, (**B**)), and p38 (**C**) mitogen-activated protein kinases in kidney homogenates of mice exposed to either air or WPS for four weeks with or without adenine treatment to induce chronic kidney disease. Mean ± SEM (n = 8). The statistical analysis encompassed a one-way analysis of variance, with subsequent application of Holm–Sidak’s multiple comparisons test.

The phospho-ERK/ERK was significantly elevated in WPS compared with air (*p* = 0.0001) and in adenine + air compared with air (*p* = 0.0001). Remarkably, the phospho-ERK/ERK was significantly increased in adenine + WPS versus either WPS (*p* < 0.0001) or adenine + air (*p* < 0.0001) (Figure 9A).

The phospho-JNK/JNK was significantly elevated in WPS compared with air (*p* < 0.05) and in adenine + air compared with air (*p* < 0.05). There was a slight but insignificant increase in phospho-JNK/JNK in adenine + WPS versus either WPS or adenine + air (Figure 9B).

The phospho-p38/p38 was significantly increased in adenine + WPS versus either WPS (*p* < 0.05) or adenine + air (*p* < 0.05) (Figure 9C).

### 2.10. Renal Histology Following WPS Exposure in Mice with CKD

**Figure 10:**
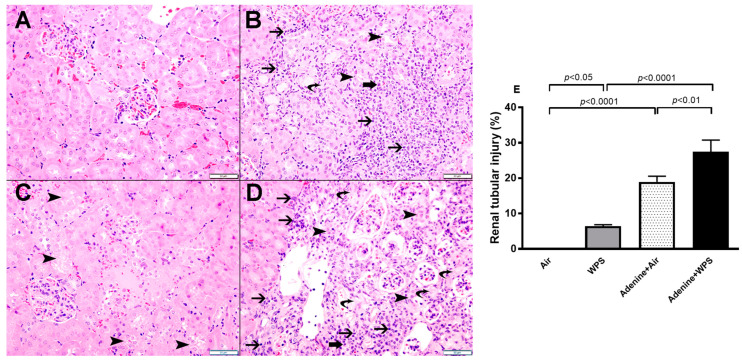
The representative light microscopy sections of kidney tissue of mice exposed to either air or waterpipe smoke (WPS) for four weeks with or without adenine treatment to induce chronic kidney disease. (**A**): Kidney section obtained from mice exposed to air showing normal kidney architecture and histology. (**B**): Kidney section obtained from adenine-treated mice and exposed to air showing tubular injury (arrowhead) and chronic inflammation with lymphocytes (thin arrow), plasma cells (thick arrow) and focal interstitial fibrosis (curved arrow). (**C**): Kidney section obtained from mice exposed to WPS for four weeks showing focal area of mild acute tubular injury (arrowhead). (**D**): Kidney section obtained from adenine-treated mice and exposed to WPS for four weeks showing tubular injury (arrowhead), chronic inflammation with lymphocytes (thin arrow), plasma cells (thick arrow) and focal interstitial fibrosis (curved arrow). (**E**): Quantification of the area of renal tubular injury (%) in the four studied groups. Data are mean ± SEM (n = 6 in each group). The statistical analysis encompassed a one-way analysis of variance, with subsequent application of Holm–Sidak’s multiple comparisons test. Scale bar in (**A**–**D**) = 50 µm.

**Figure 11:**
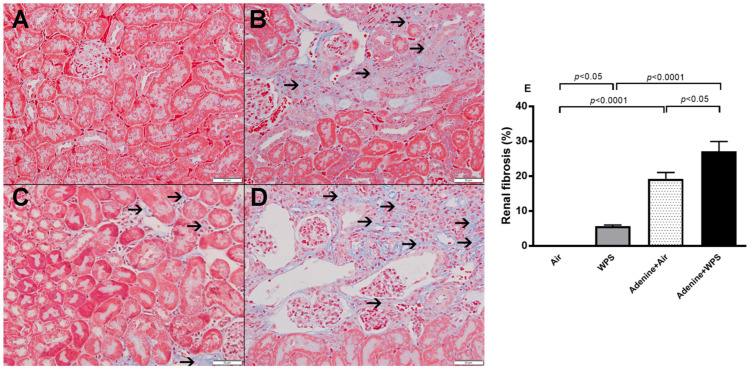
The representative images of Masson trichrome staining of kidney tissue obtained from mice exposed to either air or waterpipe smoke (WPS) for four weeks with or without adenine treatment to induce chronic kidney disease. (**A**): Kidney section obtained from mice exposed to air showing normal kidney architecture and histology. (**B**): Kidney section obtained from adenine-treated mice and exposed to air showing increase in interstitial fibrosis (thin arrow). (**C**): Kidney section obtained from mice exposed to WPS for four weeks showing focal mild interstitial fibrosis (thin arrow). (**D**): Kidney section obtained from adenine-treated mice and exposed to WPS for four weeks showing a marked increase in interstitial fibrosis (thin arrow). (**E**): Quantification of the area of fibrosis (%) in the four studied groups. Data are mean ± SEM (n = 6 in each group). The statistical analysis encompassed a one-way analysis of variance, with subsequent application of Holm–Sidak’s multiple comparisons test. Scale bar in (**A**–**D**) = 50 µm.

Light microscopy analysis of the kidney sections stained with H&E obtained from air-exposed mice shows normal kidney architecture and histology (Figure 10A). Masson trichrome stain shows no evidence of fibrosis (Figure 11A). The kidney sections collected from adenine-treated group exposed to air showed foci of tubular injury in 18.96 ± 1.583% of examined tissue areas (Figure 10B). There was a loss of brush border of proximal tubules, mixed inflammatory cells infiltration of the interstitium consisting of lymphocytes and plasma cells, and focal tubular atrophy (Figure 10B). In this group, the area of interstitial fibrosis reached 19.22 ± 1.785% of cortical tissue (Figure 11B). WPS-exposed group revealed the presence of focal tubular injury in 6.4± 0.38% of cortical tissue. The latter was in the form of tubular cells vacuolation, loss of brush border and intralumenal cast (Figure 10C). In this WPS group, Masson trichrome stain showed focal mild increase in interstitial fibrosis 5.66 ± 0.32% of cortical tissue (Figure 11C). The sections obtained from adenine + WPS group revealed the presence of foci of tubular injury with loss of brush border of proximal convoluted tubules involving 27.50 ± 3.285% of cortical tissue, mixed inflammatory cells infiltration of the interstitium consisting of lymphocytes and plasma cells and focal tubular atrophy (Figure 10D). The extent of interstitial fibrosis reached 27.04 ± 2.8% of cortical tissue (Figure 11D).

## 3. Discussion

Our study provided novel evidence that WPS exposure deteriorates kidney injury, and induced oxidative stress, inflammation, DNA damage and fibrosis in mice with adenine-induced CKD. These effects were associated with mechanisms involving NF-κB and MAPK (ERK and p38) activations. 

Smoking is a principal cause of preventable mortality worldwide, and increases the risk of developing CKDs [6,20,21,22]. The link between longer smoking duration with a higher risk of progression of CKDs is well established [6,20]. On the other hand, it has been reported that the risk of adverse kidney consequences declined with longer smoking cessation periods in former smokers [23]. It has been reported that tobacco smoking is an independent risk factor for stages 3 to 5 of CKD in women, which substantially augmented the risk approximately by 6 times for current smokers (hazard ratio: 5.7; confidence interval 95%: 2.7–12.1), but did not remain significant in past-smokers (hazard ratio: 1.6; confidence interval 95%: 0.6–4.2). However, no associations were found among men [24,25]. Experimental studies have reported that WPS inhalation causes the deterioration of kidney function, inflammation and oxidative stress [12,13]. It has also been shown that maternal exposure to WPS altered kidney function and induced oxidative stress in adult offspring rats [26]. However, little is known on the effects of WPS inhalation in mice with experimental CKD. Animal models of CKD are important and much needed to advance our understanding of the pathophysiologic complications observed in CKD [27]. A non-surgical animal model of CKD developed by the administration of adenine in diet has been reported to be a valid and reliable model to cause CKD in both rats and mice [18]. Following ingestion of adenine by mice and rats, it is metabolized to 2,8-dihydroxyadenine, that precipitates and generates tubular crystals which then injure the renal tissue [18,19].

Our data demonstrate that WPS inhalation diminished body weight compared with the air-exposed group, and that the combination of adenine and WPS further exacerbated the weight loss when compared with the adenine + air and WPS groups. Both water intake and urine output were significantly augmented in mice with adenine and exposed to WPS compared with either the adenine + air or WPS group. Similarly, while both plasma concentrations of urea and creatinine were increased, creatinine clearance decreased the in adenine + WPS when compared with adenine + air and WPS groups, confirming the exacerbation of kidney function deterioration in mice with CKD exposed to WPS. Such findings have not been reported before. It has been reported that intratracheal instillation of diesel exhaust particles (DEPs) in mice with adenine-induced CKD caused an increase in plasma concentrations of creatinine and urea and a decrease in creatinine clearance compared with healthy mice exposed to DEPs [28]. However, no difference was reported between adenine + DEPs and adenine + saline [28]. Indoxyl sulfate, a well-known protein-bound uremic toxin, is a pathogenic factor of CKD, and cardiovascular and bone diseases [29,30,31]. This uremic toxin is produced from tryptophan by the intestinal flora and is mostly eliminated via tubular secretion [31]. Here, we also found a significant increase in the concentration of indoxyl sulfate in the adenine + air group compared with air-exposed group, indicating the presence of signs of uremia. Of note, the combination of WPS with adenine induced a more significant increase in indoxyl sulfate when compared with adenine + air and WPS. Elevation of serum indoxyl sulfate has been reported to predict prognosis of CKD, and is associated with all-cause mortality and cardiovascular mortality in CKD patients [31]. Indoxyl sulfate administration has been reported to diminish superoxide scavenging activity, signifying reduced antioxidative system in the kidneys [31].

Alongside the classical biomarkers of renal injury (e.g., plasma urea and creatinine), here, we assessed the concentration and activity of some novel and more sensitive biomarkers of renal disfunction. KIM-1 is a relatively novel and sensitive biomarker of renal damage, and urinary concentrations KIM-1 have been reported to correlate with proteinuria [32]. NGAL concentration was initially used as a novel marker for acute kidney injury and we and others have validated its use as a reliable index of CKD [33,34]. NAG is a hydrolytic lysosomal enzyme found mainly in proximal tubules, and is considered a good marker of renal tubular injury [35]. Adiponectin, secreted from adipose tissue, has been reported to possess anti-inflammatory and anti-atherosclerotic actions in the healthy organism; however, in CKD, it is often elevated, and used as a biomarker of progression of CKD and a predictor of mortality [35]. It has been previously reported that KIM-1, NGAL, NAG and adiponectin increased in the urine of rats with adenine-induced CKD [33,36]. In the present study, we show for the first time that the levels of the aforementioned sensitive and specific biomarkers of kidney damage were substantially increased in mice with adenine-induced CKD and exposed to WPS compared with either the adenine + air group or WPS group.

In CKD, long-lasting and repeated acute inflammation and impaired antioxidative systems are common and worsen progressively with the degree of kidney injury [37]. Inflammation and oxidative stress play central role in the defense mechanisms against toxicants and infectious agents [37]. However, if not appropriately controlled, they may trigger various damaging actions, such as cytokine overproduction and elevations in pro-inflammatory and oxidative stress mediators [37]. In this study, inhalation of waterpipe smoke (WPS) led to a notable elevation in plasma concentrations of TNFα and IL-1β when compared to the air-exposed group. Similarly, the concentrations of TNFα and IL-1β in the plasma were heightened in the adenine + air group compared to the air group. Interestingly, the concentrations of TNFα, IL-6, and IL-1β in the plasma of the adenine + WPS group exhibited significant increases in comparison to both the adenine + air and WPS groups. Moreover, along with the plasma concentrations, compared with the air-exposed group, inhalation of WPS elevated the kidney tissue concentrations of the proinflammatory cytokines (TNFα, IL-6 and IL-1β) and the markers of oxidative stress (LPO, GSH and SOD). Likewise, in the adenine + air group, there was a substantial elevation of the above-mentioned markers of inflammation and oxidative stress compared with air-exposed mice. Moreover, these actions were markedly aggravated in mice treated with adenine and exposed to WPS compared with both WPS and adenine + air groups. It has been previously reported that exposure to diesel exhaust particles potentiates both inflammation and oxidative stress in rats with cisplatin-induced acute kidney injury and mice with adenine-induced CKD [28].

It is well-established that oxidative stress is injurious to lipids, proteins, and DNA [38,39,40]. Patients with CKD have increased DNA injury [41]. Markers for DNA damage which are commonly assessed in CKD are DNA strand breaks using the comet assay and the base modification 8-OH-dG [41]. In the present study, we quantified the DNA damage in the kidney tissue using a comet assay and by measuring the concentrations of 8-OH-dG in the urine. Our data show that both DNA damage assessed by comet assay and the concentration of 8-OH-dG in the urine were significantly increased in the WPS versus air and adenine + air versus air groups. The latter effects were exacerbated in mice treated with adenine and exposed to WPS compared with either adenine + air or WPS group.

NF-κB is a transcription factor involved in the modulation of various target genes which are linked with inflammation, oxidative stress, cell proliferation and apoptosis [42]. NF-κB is activated in both human patients with kidney diseases and animal models of renal inflammation and injury [42]. Also, the MAPK family consists of ERK, JNK, and the p38 MAPK subgroups. MAPKs play a role in transmitting signals from extracellular stimuli to nucleus, where they are frequently the decisive regulatory proteins. Sizable experimental evidence supports the concept that alterations in the cellular redox state by the initiation of an oxidative stress activate signaling pathways including several members of the MAPK family [43]. Additionally, it is well established that oxidative stress provokes the stimulation of a cascade of signaling proteins, including NF-κB and MAPKs [36,44]. This abnormal activation of NF-κB and MAPKs leads to release of inflammatory cytokines subsequently aggravating the kidney homeostasis [36,44]. Our data demonstrate that mice with adenine-induced CKD exposed to WPS exhibit a significant increase in the phospho-NF-κB/NF-κB, phospho-ERK/ERK and phospho-p38/p38 compared with those treated with either WPS or adenine + air. These effects were not observed with phospho-JNK/JNK in which there was only a significant increase in WPS compared with air and adenine + air compared with air. It has been recently reported that the expression of MAPKs is increased in the kidney of mice with adenine-induced CKD, and that the treatment with metformin mitigated this action [44]. Moreover, it has been recently reported that WPS exposure in hypertensive mice induced an overexpression of NF-κB in heart tissue compared with normotensive mice exposed to WPS or hypertensive mice exposed to air [45].

Furthermore, in line with the present biochemical and physiological findings, our histopathological data confirm that, compared with the WPS and adenine + air groups, the concomitant treatment with WPS and adenine induced more tubular injury, chronic inflammation with lymphocytes and plasma cell infiltration and interstitial fibrosis.

## 4. Materials and Methods

### 4.1. Animals and WPS Exposure

Identical numbers of six- to eight-week-old male and female C57BL/6 mice (College of Medicine and Health Sciences animal house, United Arab Emirates University, Al Ain, United Arab Emirates) weighing 25–30 g were maintained in a conventional animal house with temperature-controlled rooms (22 ± 1 °C) and kept on a 12 h light–dark cycle with a humidity of 50–60%. They had unlimited access to drinking water and commercial laboratory chow, which was composed of 8% fiber, 24% protein and 2% fat (National Feed and Flour and Marketing Co., Abu Dhabi, United Arab Emirates). The animals were kept in cages for the complete duration of the experiments, excluding 24 h before sacrifice, where they were housed in metabolic cages, to enable urine collection. The mice will be weighed at the beginning of the experiment and just before sacrifice.

A well-established animal model using adenine diet to induce CKD in mice was used [18,19,34,46,47]. To cause CKD, mice received powdered diet comprising adenine 0.2% w/w (i.e., 0.2 g adenine/100 g feed) for seven days to induce renal tubular injury [18]. Thereafter, they were kept on a 0.15% adenine diet [18] for the complete duration of the experiments (four weeks). Control mice received normal food for the same period of time. On day seven, i.e., when renal tubular injury was induced in adenine-treated mice [18], the animals were divided into four groups, as follows:

- Air group: this control group was given standard feed and exposed to air only for 30 min, 5 times/week for four weeks.
- WPS group: this group was given standard feed and exposed to WPS for 30 min, 5 times/week for four weeks.
- Adenine + air group: this group was given the same standard diet in powdered form containing 0.15% adenine and exposed to air only for 30 min, 5 times/week for four weeks.
- Adenine + WPS group: this group was given the same standard diet in powdered form containing 0.15% adenine and exposed to WPS for 30 min, 5 times/week for four weeks.

Regarding the protocol of exposure to WPS or air, mice were placed in soft restraints which were attached to the exposure tower [48]. They were exposed to WPS or air by inhalation via their noses with a nose-only exposure system (InExpose System, Scireq, Montréal, QC, Canada) [48]. Mice were exposed to WPS produced by commercially available apple-flavoured tobacco (Al Fakher, Ajman, United Arab Emirates) [48]. The tobacco was lit with an instant light charcoal disk. In compatibility with the human exposure scenario, the smoke from the waterpipe moves first via the water before it is drawn into the WPS exposure tower. The exposure method was controlled by a computerized system (InExpose System, Scireq, Canada). A computer-monitored puff was generated each minute, consisting of one puff of 2 s of WPS followed by 58 s of fresh air [48]. The daily exposure session duration to WPS was 30 min. The latter was selected from human investigations using WPS [49,50,51].

### 4.2. Blood and Kidney Collection

One day before their sacrifice, mice were transferred to metabolic cages for urine collection over a 24 h period, and its volume was measured. On the day of sacrifice, animals were anesthetized with sodium pentobarbital (60 mg/kg) given intraperitoneally. The blood was collected from the inferior vena cava in heparinized tubes, and then centrifuged at 900× *g*, at 4 °C for 15 min to separate plasma. The collected plasma was stored at −80 °C pending biochemical analyses. Mice were then sacrificed with an overdose of sodium pentobarbital. The kidneys were removed from the mice, washed with ice-cold saline, blotted with a piece of filter paper, and weighed. A small portion from the right kidney and fixed in 10% buffered formalin pending histological analysis. The rest of the right and left kidneys were individually wrapped in aluminium foil and then dipped in liquid nitrogen and kept at −80 °C, pending analysis. The preparation of kidney homogenates for the quantification of markers of oxidative stress and inflammation were carried out as described earlier [28,52].

### 4.3. Biochemical Tests in Plasma, Urine, Kidney Homogenate

The concentrations of urea in plasma and creatinine in plasma and urine were spectrophotometrically measured by means of commercially available kits (Roche Diagnostics, Indianapolis, IN, USA). Indoxyl sulfate was measured using an ELISA kit and NAG was assessed with a colorimetric kit from MyBioSource, Inc. (San Diego, CA, USA). The urinary concentration of NGAL, KIM-1 and adiponectin were measured with ELISA kits from R & D systems (Minneapolis, MN, USA). The concentrations of TNFα, IL-6 and IL-1β were quantified by means of ELISA kits acquired from R & D systems (Minneapolis, MN, USA). The NADPH-dependent membrane LPO was measured as a thiobarbituric acid reactive substance utilizing malonedialdehyde as standard (Sigma-Aldrich Fine Chemicals, St. Louis, MO, USA). The concentration of GSH (Sigma-Aldrich Fine Chemicals, St. Louis, MO, USA) and the activity of SOD (Cayman Chemicals, Ann Arbor, MI, USA) were evaluated as per to the vendor’s protocol. The concentration of 8-OH-dG in urine was assessed according to the manufacturer’s instructions provided in commercially available assay kit obtained from Cayman Chemicals (Ann Arbor, MI, USA).

### 4.4. Assessment of Kidney DNA Damage

Kidneys were removed immediately after the sacrifice, and the comet assay was executed as reported before [48,53,54]. The assessment of length of the DNA migration (viz., diameter of the nucleus plus migrated DNA) was measured by means of the image analysis Axiovision 3.1 software (Carl Zeiss, Toronto, ON, Canada) [53,54,55].

### 4.5. Western Blot Analysis

Protein expressions for total and phosphorylated NF-κB, ERK, JNK, and p38, were quantified using Western blotting techniques. Kidney tissues, collected from the mice, were snap frozen immediately with liquid nitrogen and stored at −80 °C. Later, the tissues were weighed, rinsed with saline and homogenized with lysis buffer (pH 7.4), containing NaCl (140 mM), KCl (300 mM), Triton X-100 0.5% (*v*/*v*), trizma base (10 mM), EDTA (1 mM), sodium deoxycholate 0.5% (*w*/*v*), protease and phosphatase inhibitor. The homogenates were centrifuged for 20 min at 4 °C. The supernatants were obtained and protein quantification was carried out with a Pierce bicinchoninic acid protein assay kit (Thermo Scientific, Waltham, MA, USA). A 35 µg sample of protein was electrophoretically separated by 10% sodium dodecyl sulfate polyacrylamide gel electrophoresis and then transferred onto polyvinylidene difluoride membranes. The immunoblots were then blocked with 5% non-fat milk and subsequently probed with rabbit monoclonal NF-κB p65 and phospho- NF-κB (Abcam, Hong Kong, China), ERK and phospho-ERK (Santa Cruz, Dallas, TX, USA), JNK and phospho-JNK (Santa Cruz, TX, USA) and p38 and phospho-p38 (Santa Cruz, TX, USA) antibodies, at 4 °C, overnight. After that, the blots were incubated with goat anti-rabbit IgG horseradish peroxidase conjugated secondary antibody (Abcam, Hong Kong, China) at room temperature for 2 h, and developed with a Pierce enhanced chemiluminescent plus Western blotting substrate Kit (Thermo Scientific, Waltham, MA, USA). The densitometric analysis of the protein bands was accomplished with Typhoon FLA 9500 (GE Healthcare Bio-Sciences AB, Uppsala, Sweden). Blots were then re-probed with mouse monoclonal GAPDH antibody (Abcam, Hong Kong, China) and used as a control.

### 4.6. Histopathological Analysis of the Kidneys

Formalin-fixed kidney tissues were dehydrated in increasing concentrations of ethanol, cleared in xylene and paraffin, and embedded using standard technique. Three-micrometer sections of paraffin blocks were prepared and stained with hematoxylin and eosin. The extent of renal tubular injury was expressed in percentage, as previously described by Ali et al. [19]. Moreover, kidney sections were stained for the presence of fibrosis using Masson trichrome stain following standard techniques [28,56]. Masson trichrome stains fibrous tissue blue. The percentage of blue-stained fibrotic area in each kidney section, stained with Masson trichrome stain, was evaluated and the fibrosis index was calculated and expressed as percentage according to the protocol previously reported [36,44,52].

### 4.7. Statistics

The statistical analyses were conducted using version 7 of GraphPad Prism Software. The normal distribution of the data was assessed using the Shapiro–Wilk normality test. Group comparisons were executed through one-way analysis of variance (ANOVA), with subsequent application of Holm–Sidak’s multiple comparisons test. Mean values along with the standard error of the mean (SEM) were reported for all data presented in figures and tables. Significance was determined at *p* values of ≤0.05.

## 5. Conclusions

Taken together, our data provide experimental evidence, probably for the first time, that WPS inhalation in an experimentally induced CKD model exacerbates kidney injury, oxidative stress, inflammation, DNA damage and fibrosis. These actions were linked with mechanisms involving NF-κB, ERK and p38 activations. Other possible molecular mechanisms may be involved, and will be the subject of further studies along with the assessment of lung injury and pulmonary function on the same model.

## Data Availability

The data presented in this study are available on request from the corresponding author.
